# Molecular evolution and diversity of the norovirus RNA-dependent RNA polymerase

**DOI:** 10.1038/s41598-026-40248-5

**Published:** 2026-02-14

**Authors:** Annika Flint, Maryam Jawad, Neda Nasheri

**Affiliations:** 1 Genomics Laboratory, Bureau of Microbial Hazards, Health Canada, Ottawa, ON Canada; 2 National Food Virology Reference Centre, Bureau of Microbial Hazards, Food Directorate, Health Canada, 251 Sir Frederick Banting Driveway, K1A 0K9 Ottawa, ON Canada; 3 Department of Biochemistry, Microbiology and Immunology, Faculty of Medicine, University of Ottawa, Ottawa, ON Canada

**Keywords:** Norovirus, Polymerase, Time-scaled phylogenetic analysis, Evolutionary rate, Positive selection, Negative selection, Evolution, Genetics, Microbiology

## Abstract

Human noroviruses (HuNoVs) are the leading cause of non-bacterial gastroenteritis worldwide, exhibiting extensive genetic diversity driven by recombination and mutation. The norovirus RNA-dependent RNA polymerase (RdRp), encoded by the NS7 gene, is the main driver of virus evolution by determining the mutation and recombination rates, and impacting viral fitness. Herein, we conducted spatiotemporal phylodynamic analysis on 1,094 complete RdRp amino acid sequences from genogroups GI and GII collected between 1972 and 2024 to investigate molecular evolution, amino acid variation, evolutionary rates, and selection pressures. Time-scaled phylogenetic analysis revealed that GI polymerases diverged into three major lineages since the year ~ 1630, while GII polymerases formed four lineages, including a distinct cluster for P16. Genotype distribution was dominated by GII.P16 (43%) and GII.P31 (20%), both historically associated with GII.4 capsids. Amino acid substitutions were rare to non-existent within conserved motifs (A–G), indicating strong structural and functional constraints. Evolutionary rate estimates showed GII polymerases evolve faster (mean 1.5 × 10⁻³ substitutions/site/year) than GI (mean 3.8 × 10⁻⁴ substitutions/site/year), though overall RdRp evolves more slowly than the major capsid protein VP1. Selection analyses demonstrated pervasive purifying selection, with limited evidence of positive selection at codons outside of the conserved motifs. These findings highlight the high conservation of RdRp, its slower evolutionary dynamics compared to capsid gene, and its role in norovirus diversification, supporting its relevance as a target for antiviral development.

**Supplementary Information:**

The online version contains supplementary material available at 10.1038/s41598-026-40248-5.

## Introduction

Human norovirus (HuNoV) is the major cause of non-bacterial gastroenteritis worldwide, and it is estimated to cause 685 million incidents and 212,000 deaths annually^1^. Noroviruses belong to the *Caliciviridae family* and are non-enveloped, single-stranded, positive-sense RNA viruses possessing a genome of approximately 7.5 kb. The genome is covalently linked to VPg and consists of three open reading frames (ORFs); ORF1 encodes a polyprotein that generates six non-structural proteins, including RNA-dependent RNA Polymerase (RdRp), which is coded by the NS7 gene^2^, ORF2 encodes the major capsid protein (VP1), which plays a vital role in encapsulating the genome, and ORF3 encodes the minor capsid protein (VP2)^2,3^. ^4,5^Noroviruses are categorized into ten genogroups (GI to GX) and further into 49 genotypes based on the diversity of the capsid VP1 gene^4^. Due to the immense diversity of noroviruses and the high degree of recombination, dual typing of VP1 and RdRp sequences was proposed for genotyping noroviruses. To date at least 14 GI P-types and 37 GII P-types have been distinguished^4^.

The RdRp of noroviruses is a critical enzyme involved in the norovirus genome’s transcription and replication processes^6^. The RdRp is comprised of approximately 510 amino acids and is described as a partially closed right hand containing fingers, thumb, and palm subdomains^6^. The fingers and thumb form a channel that is essential for template binding and interactions with the incoming nucleoside triphosphates (NTPs)^6^. The palm subdomain, positioned between the fingers and thumb subdomains, contains the most structurally conserved elements including the template-recognizing motif and the catalytic site^7,8^.

There are seven conserved domains (A to G) within the norovirus virus RdRp: motif A is mainly in the palm subdomain, coordinates metal ions for catalysis and NTP binding; motif B is involved in discrimination between NTPs and deoxy NTPs, and template positioning; motif C houses a conserved Gly-Asp-Asp (GDD) motif involved in catalysis; motif D enables the movement of the thumb subdomain during elongation and plays a critical role in selecting NTPs^6–8^. At the intersection of the palm and the thumb subdomain is motif E, which is involved in template binding, motif F interacts with the phosphate group of NTPs, and lastly, motif G forms part of the template entrance channel and interacts with the priming NTPs^7,8^.

Viral RdRps interact with the host factors for efficient genome replication, and changes in these proteins affect viral fitness and determine strain emergence and host tropism^9^. Analyzing the variations in the polymerase gene of noroviruses is a valuable approach for developing antivirals and understanding viral evolution and transmission. The replication rate of a virus is a determinant of viral fitness, since viruses with increased replication rate can produce more copies of their genome, which would result in more variants even if the RdRp error rate remains constant^2^. Therefore studying the variations in viral polymerase allows for understanding viral fitness, explains evolutionary relationships among different variants, and strain emergence. This study aims to provide a comprehensive overview of the evolution of the norovirus RdRp gene by conducting time-scaled phylogenetic analysis, amino acid substitutions and mutation rates analyses and identifying positive and negative selection at the RdRp level in GI and GII.

## Materials and methods

### Data curation

Full-length nucleotide and amino acid sequences of norovirus GI and GII RdRp gene NS7 were obtained from GenBank (as of March 2024). Strains possessing multiple undetermined nucleotides (denoted as “N”) and amino acids (denoted as “X”) were excluded. P-types that had fewer than ten sequences were also excluded from our analysis. A total of 968 norovirus GII RdRp sequences were included in our analysis, encompassing 10 GII P-types : 12 P3, 16 P6, 12 P7, 15 P8, 76 P12, 49 P15, 413 P16, 60 P17, 118 P21, and 197 P31 sequences. A total of 126 GI RdRp sequences were retrieved encompassing 7 GI P-types :12 P1, 11 P2, 34 P3, 25 P4, 12 P6,14 P11, and 18 P13 sequences. For GenBank entries without RdRp annotation, RdRp sequences were identified by BLASTn (v2.12.0+) using default parameters and perc identity 70^10,11^. GenBank accessions used in this study are listed in Supplementary Tables 1 and 2.

### Time-scaled phylogenetic trees

RdRp amino acid sequences of each polymerase type were aligned with MUSCLE (v5.1) using default settings. IQ-Tree (v3.0.1)^12,13^ using ModelFinder was used to determine the best-fit substitution model (JTT + I+G4). Time-scaled phylogenetic reconstruction was performed with BEAST (v2.7.7)^14^ using a Bayesian Markov Chain Monte Carlo (MCMC) method with 50 million iterations (sampling every 1000 steps) for GI and 450 million iterations (sampling every 5000 steps) for GII. BEAST was run using a relaxed molecular clock with log normal distribution and coalescent Bayesian skyline tree prior. Convergence and effective sample sizes (ESSs) were analyzed using Tracer (v1.7.2)^15^ and parameters with ESSs > 200 were accepted. Node credibility was assessed based on 95% highest posterior density (HPD) intervals. The TreeAnnotator package within BEAST was used to create Maximum Clade Credibility trees using 10% or 15% burn-in for GI and GII RdRp types, respectively. Data visualization of phylogenetic trees was performed using iTol (v6.9)^16^.

### Evolutionary rate analysis

Evolutionary rates for each RdRp type were estimated in BEAST (v2.7.7) using 50 million iterations with sampling every 1000 steps. Only RdRp types that achieved convergence and effective sample sizes (ESS > 200) were included for rate estimation.

### Amino acid substitution analysis

RdRp amino acid sequences of each polymerase type were aligned with MUSCLE (v5.1)^17^ using the default settings. A consensus sequence for each alignment was generated using the SummaryInfo.dumb_consensus function in Biopython (github.com/biopython). Mutations were identified using a custom Python script that compared each amino acid position in the alignment to the consensus sequence, with differences recorded as position-specific mutations.

### Positive and negative selection analysis

RdRp nucleotide sequences of each polymerase type were aligned with MUSCLE (v5.1) using default settings^17^. Alignments were visually inspected to confirm reading frame and duplicate sequences were removed with HyPhy (Hypothesis Testing using Phylogenies) (v2.5.76)^18^and the remove-duplicates command. The best-fit nucleotide substitution model for each polymerase type alignment was determined using ModelFinder in IQ-TREE (v3.0.1^12,13^). Maximum likelihood (ML) phylogenetic trees based on the best-fit substitution model were constructed with IQ-Tree using the aligned sequences, codon sequence type option (-st CODON) and 1,000 bootstrap replicates (-bb 1000). Positive selection analysis was performed with HyPhy (v2.5.76)^18^using FEL (Fixed Effects Likelihood), FUBAR (Fast, Unconstrained Bayesian AppRoximation), MEME (Mixed Effects Model of Evolution), and BUSTED (Branch-site Unrestricted Statistical Test for Episodic Diversification) models. For FEL, sites with dN/dS > 1 and *p* < 0.1 were considered under positive selection, whereas sites with dN/dS < 1 and *p* < 0.1 were considered under negative selection. For FUBAR, sites with posterior probability > 0.9 were considered positively or negatively selected. For MEME and BUSTED, a significance threshold of *p* < 0.1 was used to identify episodic diversifying selection.

### RdRp protein modeling

Homology models of the norovirus RdRp were generated using the crystal structure of the GII.P4 isolate Sydney348/97O/AU (PDB ID: 4LQ3) as the structural template. Structural modeling was performed with MODELLER v10.8^19^ using aligned consensus amino acid sequences for RdRp P-types. Model quality was assessed by Ramachandran plot analysis using the Ramplot server^20^. Across the P16, P21, and P31 RdRp models, 97.8–98.4% of residues were located in favoured regions, 1.0–1.8% in allowed regions, and 0.4–0.6% in disallowed regions. The first five N-terminal residues of each model were constructed ab initio due to their absence in the template structure (PDB ID: 4LQ3). Models were visualized using pymol-open-source (v3.1.0 [github.com/schrodinger/pymol-open-source]) with the 5 N-terminal amino acids excluded from final structural visualizations.

## Results

### Description of sequences

**Fig. 1:**
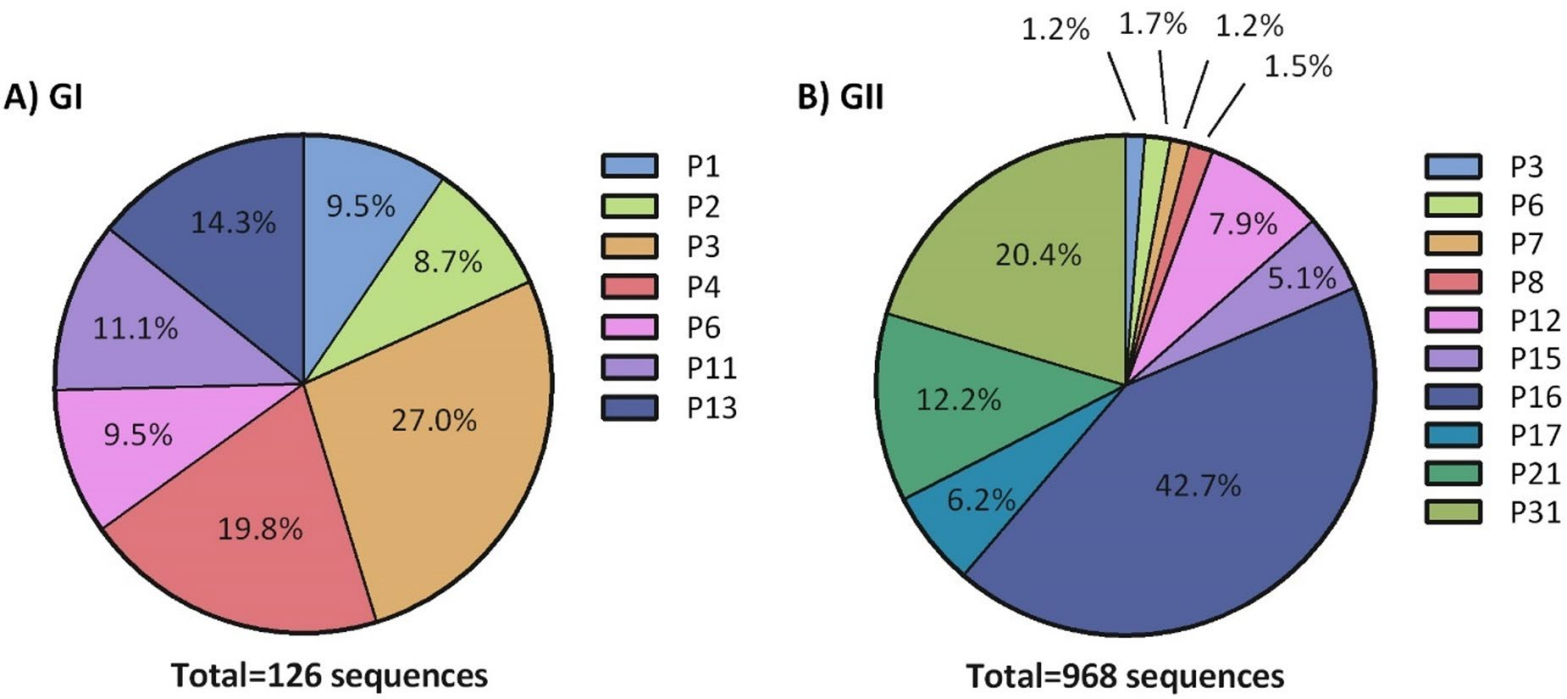
The P-type distribution for the RdRp (NS7) sequences analyzed in this study. **A**) GI sequences *n* = 130 **B**) GII sequences *n* = 1027.

To gain a comprehensive understanding of the current evolutionary trend of HuNoV GI and GII, a total of 1,094 complete RdRp (NS7) sequences from 1972 to 2024 were obtained from GenBank. The majority of the sequences belong to GII (968), and 126 belong to GI. The genotype distribution for both genogroups are demonstrated in Fig. 1a, and b. For GI polymerases, the majority of P-types belong to P3 (27%), P4 (20%) and P13 (14%). For GII polymerases, P16 (43%) had the highest prevalence followed by P31 (20%). Interestingly, both of these P-types have been associated with GII.4 genotype, which was the dominant genotype for decades^21^. On the other hand, P3, P6, P7, and P8 altogether comprised only 5.7% of the GII P-types.

### Time-scaled phylogenetic analysis

**Fig. 2:**
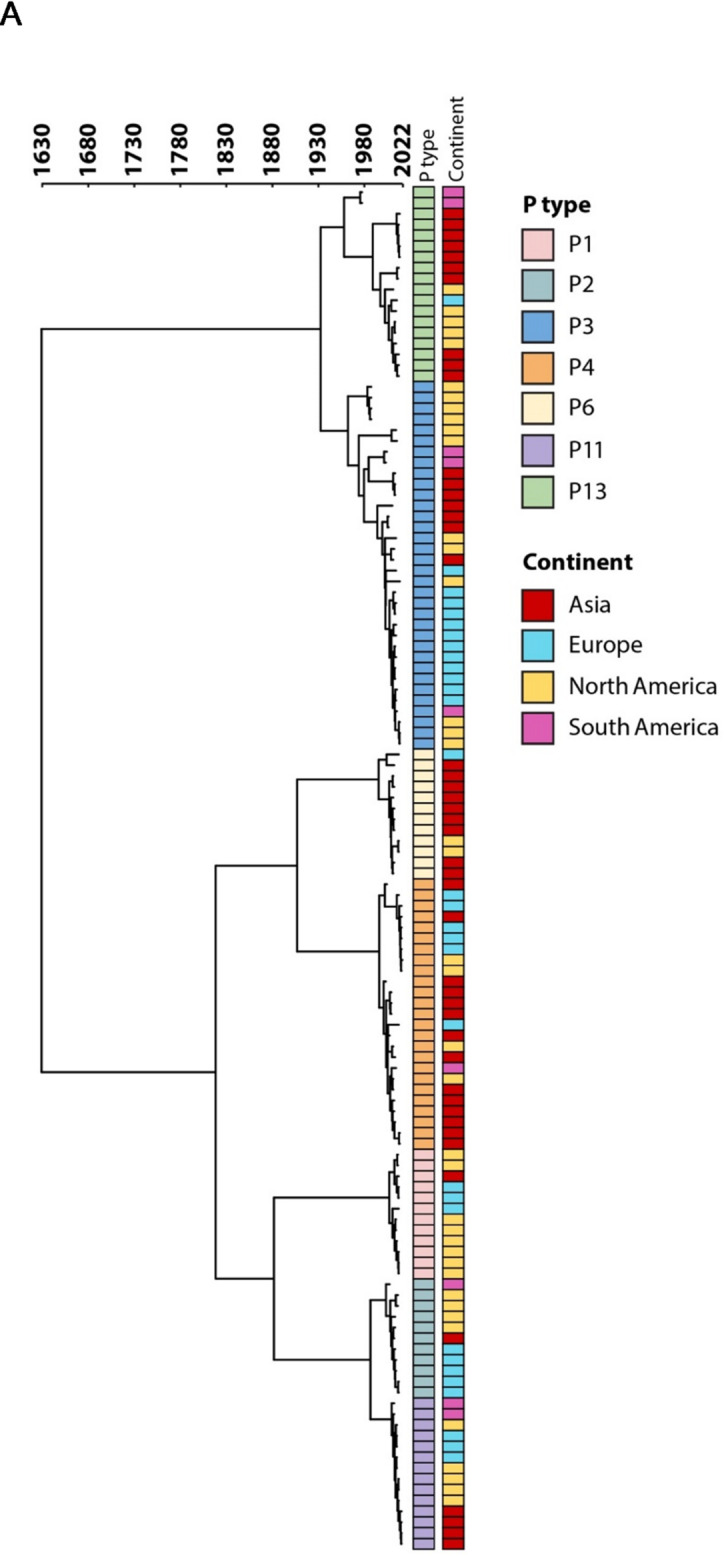

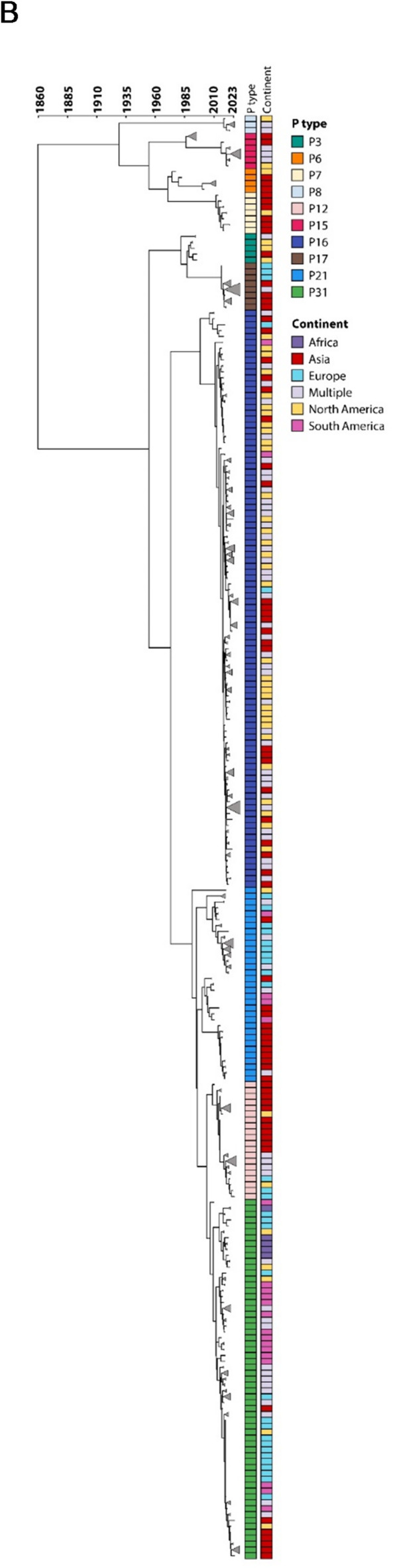
Time-scaled phylogeny of norovirus RdRp types. Multi-protein alignments were generated using MUSCLE. BEAST (v2.7.7) using a Bayesian Markov Chain Monte Carlo (MCMC) method was used to construct the maximum clade credibility tree. The scale axis is represented in years and only RdRp types that had more than 10 sequences were included in the analysis. (**A**) GI RdRp sequences were obtained from GenBank (P1 = 14, P2 = 11, P3 = 34, P4 = 26, P6 = 13, P11 = 14, P13 = 18). (**B**) GII RdRp sequences were obtained from GenBank (P3 = 15, P6 = 19, P7 = 12, P8 = 15, P12 = 76, P15 = 49, P16 = 449, P17 = 61, P21 = 125, P31 = 206) and clades are collapsed at an average branch length to tip distance of < 5 and are shown as proportional triangles by RdRp type.

We next proceeded with time-scaled phylogenetic analysis incorporating geographic distribution, collection date, and P type for each genogroup. As demonstrated in Fig. 2a, the common ancestor of the obtained HuNoV GI RdRp is likely emerged around the year 1630 and diverged into three lineages: lineage 1 that evolved in 1930s (P3, P13; 95% HPD 1900–1962), lineage 2 that evolved in 1880s (P6, P4; 95% HPD 1843–1976) and lineage 3 evolved in 1850s (P1, P2, and P11; 95% HPD 1802–1965). Although we did not obtain any RdRp GI sequences from Africa and sequences from South America are under-represented, there is no clear geographical clustering in the sequences.

The Bayesian phylogenetic tree of the RdRp gene for the GII sequences included in this study indicates that the common ancestor is likely emerged prior to 1860 and evolved into 4 lineages: Lineage 1 diverged in 1930s (P6, P7, P8, P15; 95% HPD 1875–1958), lineage 2 evolved in 1970s (P3, P17; 95% HPD 1907–1975), Lineage 3 diverged in 1990 and only contains P16 (95% HPD 1982–2002). Lineage 4 diverged in late 1980s and includes (P12, P21 and P31; 95% HPD 1975–1993) (Fig. 2b). Similar to HuNoV GI types, there is no distinct geographical clustering, which demonstrates wide global spread and diversification without regional confinement. As expected the majority of the sequences belonged to P31 and P16 as they were responsible for the majority of the cases over the past two decades. In contrast, P3, P6, and P7 types were not observed in the recent sequences. It is noteworthy that multiple clusters were observed within each P type, and this clustering is more chronologically distinct within P15, P16, P21 and P31. This information could be used to potentially update the classification of norovirus P-types.

### Variations within RdRp gene

**Fig. 3:**
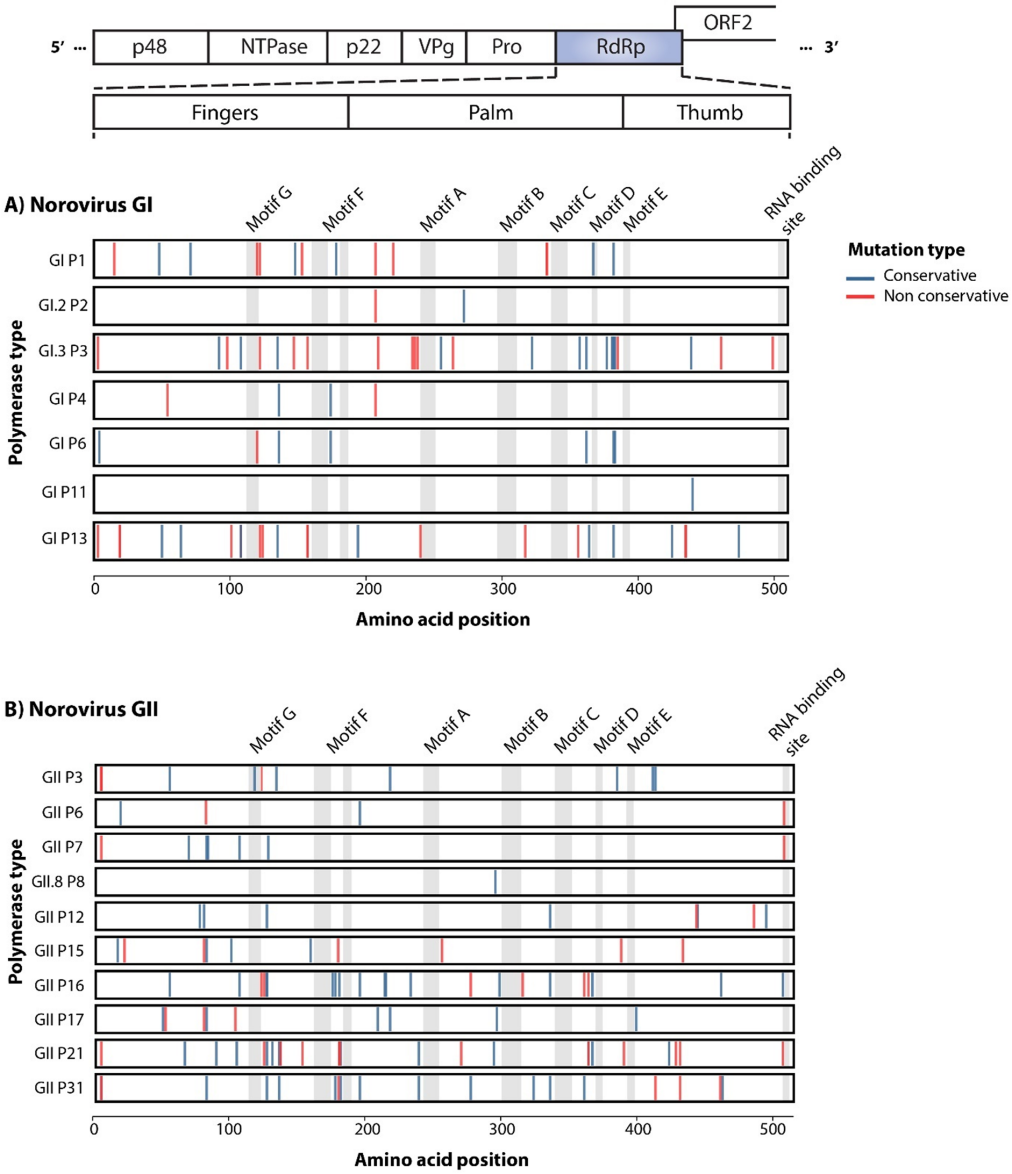
Distribution of amino acid substitutions in norovirus polymerase types. (**A**) Norovirus GI and (**B**) GII substitutions occurring at ≥ 5% frequency and present in at least two sequences are shown. Mutations are mapped to amino acid positions (lines) within the fingers, palm, thumb or conserved motif regions of RdRp (shaded gray). Mutations are coloured as blue (conservative changes) or red (non-conservative changes) lines relative to the consensus sequence.

We next examined the nonsynonymous mutations in RdRp across P types for both HuNoV GI and GII (Supplementary Table 3). The substitutions that occurred at 5% or higher are demonstrated in Fig. 3. Both biochemically conservative (blue) and non conservative (red) substitutions are fewer in the GI RdRp (93) compared to the GII RdRp (1028), which could be partly explained by the difference in number of obtained sequences. In the GI sequences P6 had the highest number of changes (63) throughout the protein while P11 had only one conservative substitution in the thumb domain (Supplemental Table 3).

For the GII RdRp protein, the highest number of substitutions was observed in P31 (300) while P17 had the fewest (18) (Supplemental Table 3). While clustering of substitutions were not observed for any of the genogroups, the functional motifs had the least number of substitutions (Fig. 3A and B). For GII, a few substitutions were observed in the functional motifs, including GII.P3 in Motif G, as well as GII.P6, GII.P7, GII.P16 and GII P21 at the RNA binding site (Fig. 3B).

**Fig. 4:**
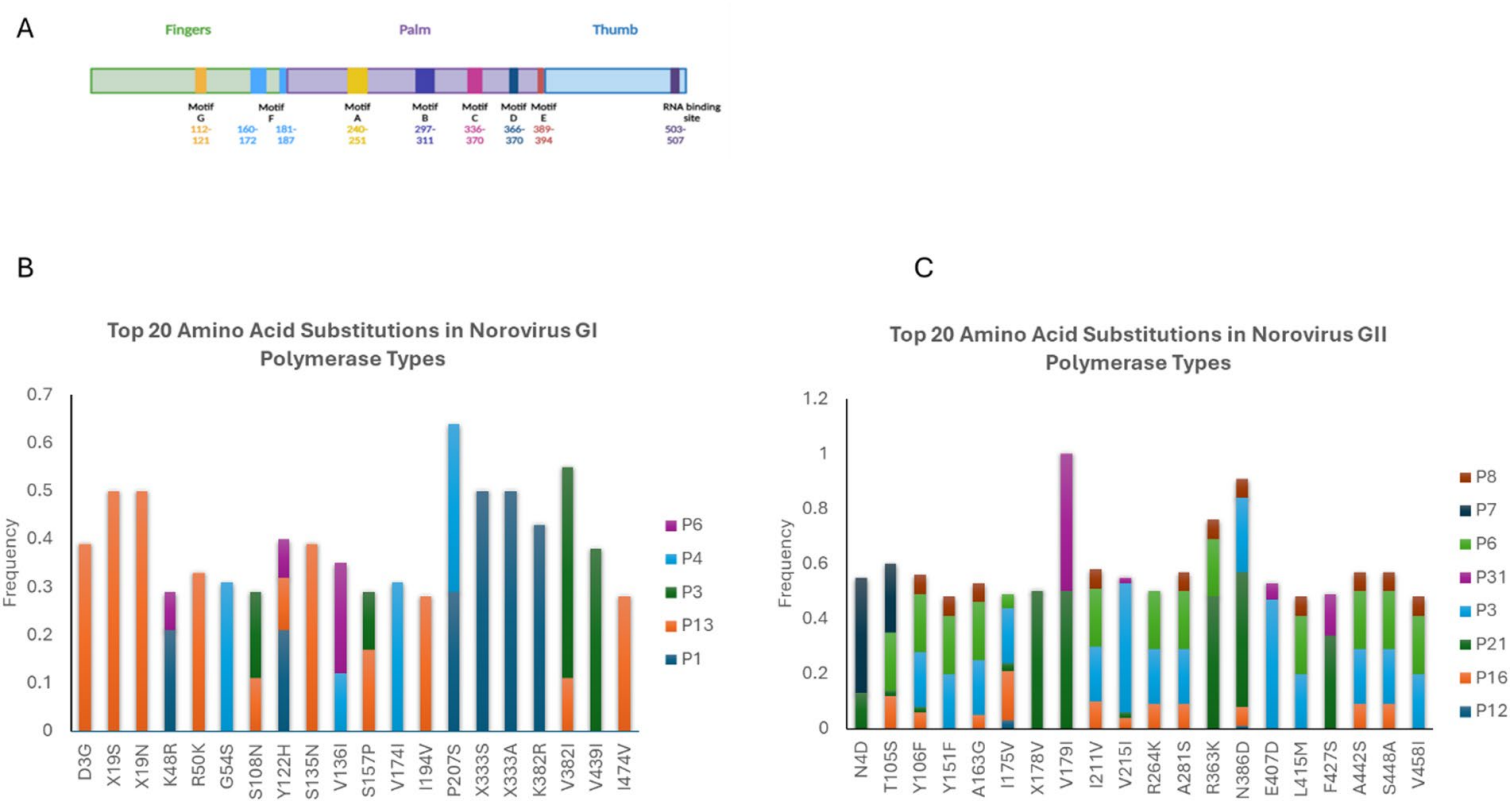
The most common 20 amino acid substitution in the RdRp protein. **A**) The scheme for RdRp protein with the fingers, palm, and thumb subdomains and the functional motifs are demonstrated. **B**) The top 20 amino acid substitutions in GI with their occurrence frequency and genotypes. **C**) The top 20 amino acid substitutions in GII with their occurrence frequency and genotypes.

We next zoomed in for the most common 20 amino acid changes in the RdRp protein and as shown in Fig. 4b, the majority of the changes (11) for the GI sequences are within the fingers domain and no change was observed with the motifs. For the GII RdRp protein, the most common substitutions occur outside of the functional motifs with the exception of the A163G change, which falls within motif F in the finger domain (Fig. 4c). This change is observed in four polymerase types, P16, P3, P6 and P8. It is noteworthy that alanine and glycine are both small non-polar amino acids and this change is not considered a biochemically drastic substitution, however, it can be structurally impactful.

Furthermore, we have identified a number of recurrent and reversible amino acid changes in the RdRp and a few of them have been recently reported for GII.P17^22^, these changes include position 81 of the RdRp (1273 of the non-structural protein), where changes such as N↔S, S↔G, T↔N, and T↔S have been observed for P3, P6, P7, P15, P16, P17, and P31 (Supplementary Table 3). The same is true for position 293 (1485 of the non-structural protein), where changes such as A↔V, T↔A, T↔S, and S↔A have been observed for P3, P16, P17, P21, and P31 (supplementary Table 3). These recurrent and reversible mutations could indicate convergent evolution across different polymerase types.

### Evolutionary rate analysis

When all RdRp P types were analyzed together within each genogroup, the mean evolutionary rate was estimated at 3.8 × 10^− 4^ amino acid substitutions per site per year (95% HPD: 2.2 × 10⁻⁴- 5.7 × 10⁻⁴ substitutions /site/year) for GI and 1.5 × 10^− 3^ substitutions per site per year (95% HPD: 1.2 × 10⁻³ − 1.8 × 10⁻³ substitutions /site/year) for GII. These results suggest that GI and GII have significantly different mean evolutionary rates, with GII RdRp evolving faster than GI.

**Fig. 5:**
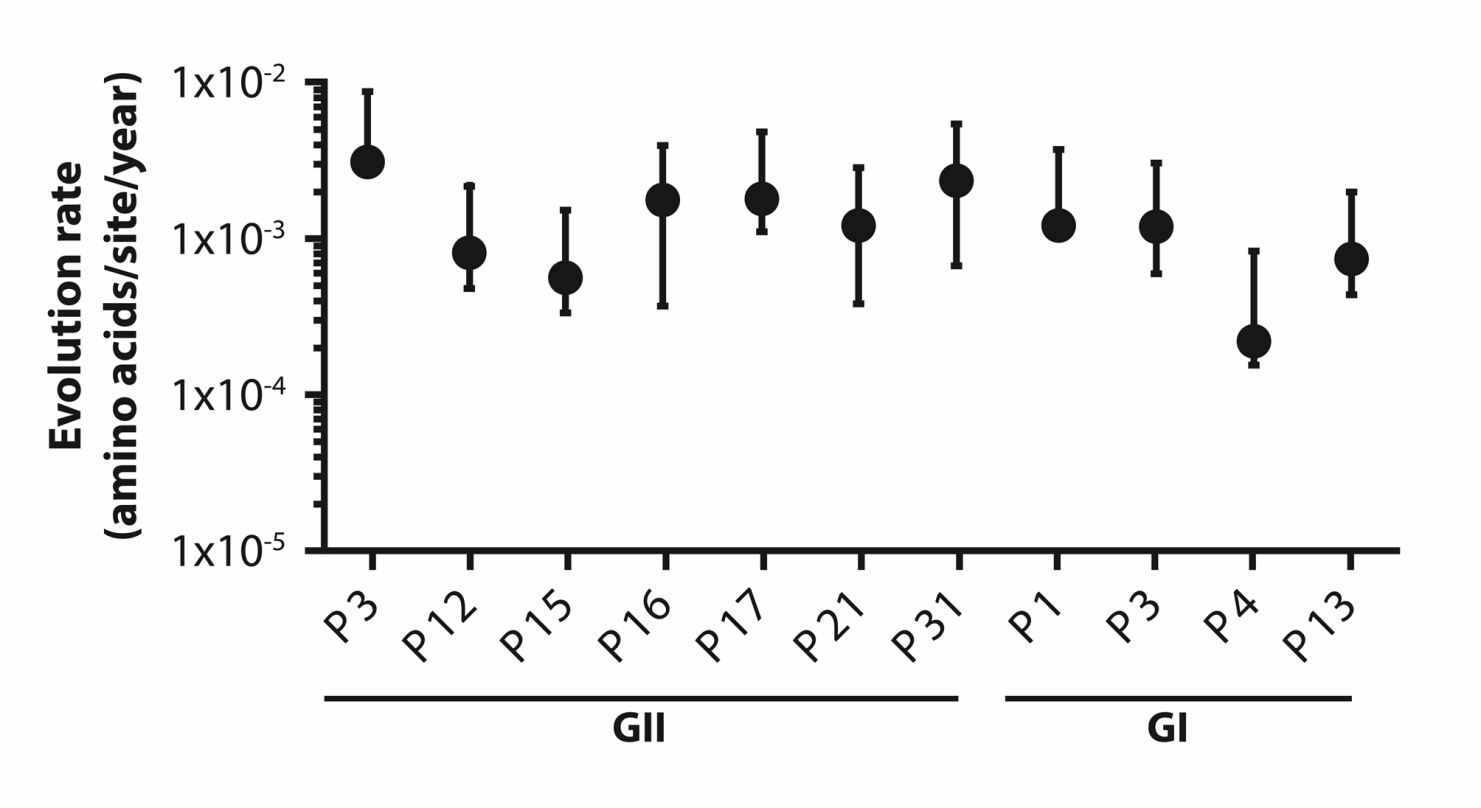
Estimates of evolution rates for GII and GI RdRp types. Rates were estimated in BEAST (v2.7.7) using a Bayesian MCMC approach on MUSCLE-aligned amino acid sequences. Mean substitution rates (amino acid substitutions per site per year) with 95% HPD intervals are shown. Only RdRp types with ESS > 200 after 50 million iterations (sampled every 1000 steps) are included.

Given these differences between genogroups, we next investigated whether similar variation occurred among the individual P types within each genogroup. Previous analyses of the VP1 gene have shown genotype-specific differences in the HuNoV evolutionary rate^3,23,24^. Thus, we sought to determine whether such heterogeneity also exists in the NS7 (RdRp) gene. As shown in Fig. 5 and Table S4, the GII P types (P16, P17, P21, P31) have evolutionary rates significantly different from GI P4 as indicated by non-overlapping 95% HPD intervals. Within genogroups, however, the 95% HPD intervals overlap among most P types (Fig. 5 and supplementary Table 4), suggesting only small differences in the rate of evolution among the P types studied. For GI polymerases, P4 had the slowest evolutionary rate with a mean of 2.2 × 10^− 4^ substitutions/site/year, while P1 and P3 had similarly higher rates, each with a mean of 1.2 × 10^− 3^ substitutions/site/year. For GII RdRps, P15 had the slowest evolutionary rate at a mean of 5.6 × 10^− 4^ substitutions/site/year while P3 and P31 had the highest evolutionary rates, with means of approximately 3.1 and 2.35 × 10^− 3^ substitutions /site/year, respectively.

### Positive selection analysis

Positive or diversifying selection, which indicates that mutations are favoured for a single site are usually assessed from the dN/dS (nonsynonymous vs. synonymous substitution) ratio. If dN/dS > 1, it indicates the site is under selective pressure and nonsynonymous (coding) mutations are favoured. In contrast, dN/dS < 1 suggests that the site is conserved and non-coding mutations are unfavoured, which imply negative or purifying selection^25,26^. We used four models; FEL, FUBAR, MEME, and BUSTED for predicting positive selection sites for each P Type (Table 1). The latter model does not provide the specific selection sites, nonetheless could be used for screening for positive selection. Across all norovirus polymerase types, most codons were inferred to be under purifying (negative) selection, with only a small number of sites showing evidence of pervasive diversifying (positive) selection by FEL or FUBAR (Table 1). MEME identified a limited number of codons subject to episodic diversifying selection. At the gene-wide level, BUSTED detected significant evidence of episodic diversifying selection in a subset of polymerase types, specifically GII.P16, GII.P21, GII.P31 and GI.P13. Polymerase types represented by fewer sequences have reduced statistical power, limiting detection of significant selection. Overall, these results suggest that RdRp sequences are predominantly subject to strong purifying selection to maintain essential structure and function of the enzyme.

**Table 1:** Positive selection analyses of Norovirus RdRp sequences. The number of codons showing diversifying (positive) or purifying (negative) selection, as well as evidence for gene-wide episodic diversifying selection are shown. All analyses were performed using HyPhy. Significance thresholds were set at *p* < 0.1 for FEL, MEME, and BUSTED, and posterior probability > 0.9 for FUBAR.

Genogroup	Ptype	Sequences (#)	Total codons	Noninvariant codons	Site model			Gene-wide test
					FEL	FUBAR	MEME	BUSTED
					Pos: Neg	Pos: Neg	Significant sites	Episodic Selection
GII	P3	12	510	144	0:59	0:96	4	no
GII	P6	16	510	399	1:300	1:340	0	no
GII	P7	12	510	317	0:163	0:289	3	no
GII	P8	15	510	354	0:239	1:252	1	no
GII	P12	76	510	248	1:110	0:202	2	no
GII	P15	49	510	254	0:97	1:224	2	no
GII	P16	413	510	478	2:416	1:457	11	yes
GII	P17	60	510	128	0:21	0:56	4	no
GII	P21	118	510	459	1:294	2:349	6	yes
GII	P31	197	510	480	2:422	1:456	14	yes
GI	P1	14	508	259	0:147	0:239	1	no
GI	P2	11	508	227	0:118	0:212	3	no
GI	P3	34	510	367	0:195	0:344	3	no
GI	P4	26	508	250	0:100	0:233	3	no
GI	P6	13	508	335	0:233	0:278	1	no
GI	P11	14	508	83	0:20	0:56	0	no
GI	P13	18	510	311	0:160	0:281	1	yes

Polymerase types represented by more than 20 sequences were considered for further analysis. Those showing positive selection in two or more HyPhy tests (Table 1) were further examined to identify codon positions under diversifying selection (Table 2). For P6, codon #503 was identified by both FEL and FUBAR. For P12, codon #490 was identified by both FEL and MEME, for P16, codon #427 was identified by FEL, FUBAR, and MEME, and for P21, codon #267 was identified by FEL, FUBAR and MEME. Finally for P31 codon #503 was identified by FEL, FUBAR and MEME. Among codons inferred to be under diversifying selection, nearly all were positioned outside the conserved RdRp motifs, consistent with structural and functional constraints on the polymerase. Only codon 503 in GII.P16 and GII.P31 was located within the RNA-binding site.

**Table 2:** Positive selection sites in Norovirus polymerase by type. Polymerase types with evidence of positive selection in two or more hyphy analyses (FEL, FUBAR, MEME) are shown. Codon positions are indicated for each polymerase type.

Genogroup	Ptype	FEL codons	FUBAR codons	MEME codons
GII	P12	490	-	439, 490
GII	P15	-	99	135, 179
GII	P16	81, 427	427	1, 59, 178, 212, 257, 274, 323, 382, 416, 427, 430
GII	P21	267	267, 360	54, 55, 94, 211, 267, 363
GII	P31	151, 503	503	18, 55, 81, 103, 107, 179, 219, 320, 327, 399, 435, 491, 501, 503

**Fig. 6:**
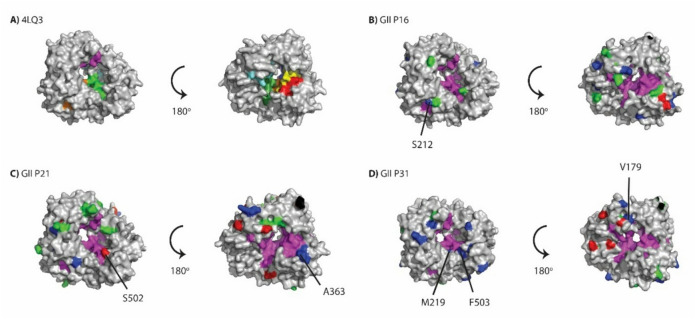
Structural modeling of GII P16, P21 and P31 RdRp proteins. (**A**) Crystal structure of GII.P4 isolate Sydney348/97O/AU (PDB ID: 4LQ3). Functional motifs A-G and RNA binding motif are shown. Motif A, yellow; motif B, blue; motif C, orange; motif D, red; motif E, dark green; motif F/F2, cyan; motif G, purple; RNA binding site, green. Structural models for (**B**) P16, (**C**) P21 and (**D**) P31. Positive selection sites identified by FEL, FUBAR or MEME are coloured blue. Conservative and non-conservative amino acid mutations (frequency > 5%) are coloured green and red, respectively. Motifs A-G and RNA binding site are coloured purple.

We next mapped positive selections sites as well as biochemically conservative and non-conservative amino acid changes in GII.P16, GII. P21 and GII.P31 on the 3D crystal structure of GII RdRp, as these had the highest numbers of amino acid substitutions and sites under positive selection, to examine the locations of these changes relative to the functional motifs (Fig. 6). For P16, S212 has close proximity to Motif C (Fig. 6B) and A312 is close to motif B and A. Also H121 is close to motif F and G along with H123 which is close to motif G but not visible in Fig. 6B. For P21 R363 is close to motif A and S502 has a close proximity to the RNA binding site (Fig. 6C). For P31, V179 is close to motif F, M219 is close to motifs C, E, and RNA binding site (Fig. 6D). Lastly F503 has a close proximity to the motif E and the RNA binding site (Fig. 6D).

## Discussion

Norovirus RdRp is a key driver of virus evolution by impacting viral fitness, recombination, and strain emergence^27,28^. Norovirus RdRp must interact with the necessary host factors for viral replication, lacks proofreading ability which enables rapid genetic diversification, and allows template switching and recombination. These traits impact the overall viral fitness^25^. Since the functions of RdRps are unique and highly conserved among RNA viruses, many attempts for developing antivirals are focused on these enzymes^6,7^. Although changes in RdRp might not directly affect the norovirus antigenicity, they can impact the spread and emergence of new strains worldwide^29^. It has been hypothesized that the differences in the error and replication rates could affect transmission at the population level. Therefore it is crucial to study the phylodynamics of norovirus RdRp to understand molecular evolution, strain emergence and predominance.

Herein, we conducted spatiotemporal phylodynamic analysis on 1,094 complete RdRp sequences from norovirus genogroup I (GI) and GII. The majority of the polymerases in this study belong to GII viruses and among them the genotype P16 had the highest prevalence followed by P31. Both of these P types have been associated with the GII.4 genotype, which was the predominant genotype for decades^29^and therefore over-represented in our dataset. It is noteworthy that the sampling for this study occurred prior to the recent predominance of GII.17 as of 2024^30^.

Our time-scaled phylogenetic analysis revealed that the common ancestor of the norovirus GI polymerases emerged prior to the year 1630, which is inline with a report that estimated that the common ancestor evolved around 500 years ago^31^. Furthermore, our analysis have shown that the GII polymerases evolved prior to 1860, which is consistent with another study that estimated their divergence around 1731^32^. Unlike GII polymerases, no distinct predominant P-type was observed for GI polymerases, and the three clusters that were identified here, have been reported previously. Close proximity between GII P6, 7, 8, and 15 in lineage 1, as well as between GII P3 and P17 in lineage 2, and between P31 and P21 in lineage 3, which are observed in our study, and have also been reported^32^. However since our work includes more recent sequences, we have identified that GII P16 forms a separate cluster (lineage 4). This polymerase was initially associated with GII.2 and GII.4 capsids^33,34^but more recently association with multiple other capsids, including GII.1, GII.3, GII. 5, GII.12, and GII.13 have been reported^35,36^. Lack of geographical clustering in norovirus phylogenetic analysis suggests that noroviruses can spread widely and rapidly, likely due to high transmissibility and global travel, without strong regional genetic differentiation^37^. This phenomenon has been observed for other human viruses causing acute infection such as influenza A(H1N1)^38^.

Furthermore, several lines of evidence suggest that GII17[P17], is on the rise globally, and a recent study identified 4 chronological clusters using the VP1 gene of the GII17 sequences^22^, interestingly, we also identified 4 clusters using the RdRp of the P17 sequences.

Several studies have reported differences in the evolutionary rate among different norovirus genotypes could account for the emergence and predominance of certain viruses^29,32,37^. Herein, we assessed the evolutionary rates based on amino acid replacement, as opposed to nucleotide substitution, as this provides more biologically relevant information on functional impacts. While, it could be expected that this method provides evolutionary rates that are slightly lower than the rates estimated from nucleotide substitutions, it provides complementary and biologically relevant insight with nucleotide-based rate substitutions. Our analyses demonstrated that the overall substitution rate for the RdRp gene was slower than the average rate for VP1 and the evolution for certain P types is slower than the average rates that has been previously reported for the genome-wide human noroviruses (1.4–5.4 × 10^− 3^ substitutions/site/year)^33^: the evolution rate for GI P4 estimated at 2.2 × 10^− 4^ and for GII P12 and P15 estimated at 8.1 × 10^− 4^ and 5.6 × 10^− 4^ substitutions/site/year, respectively. Overall, our observations demonstrate that the amino acid substitutions in RdRp occur at a slower pace compared to the VP1 protein and are non-existent in the functional motifs, implying that RdRp is more conserved compared to VP1. This is not surprising as RdRp is not the target of neutralizing antibodies and thus is under less immune selection pressure from the host compared to VP1. This is contrary to the report that found noroviruses evolve at similar rate across their genome^33^. Furthermore, our study demonstrates that across all norovirus polymerase types, most codons were under negative selection, with only a small number of sites showing evidence of positive selection, which all fall outside of the functional conserved motifs. Once we mapped the selections sites on the 3D structure of the RdRp, we discovered some of the codons under positive selection, are in close proximity of the functional motifs, which could impact the RdRp activity.

Furthermore, the more recent P types such as P17 and P16 did not show a higher evolutionary rate (similarly 1.8 × 10^− 3^ substitutions/site/year) compared to the more established P types such as P31 (2.4 × 10^− 3^ substitutions/site/year). This observation has also been documented for the evolution of the VP1 gene, that the newly emerged genotypes do not necessarily evolve at a higher rate^33^. Also our data demonstrate that the overall average substitution rate for the GII polymerases is faster than the GI polymerases, with the average rate for GI polymerases being 3.8 × 10^− 4^ substitutions/site/year while the average rate for GII polymerases being 1.5 × 10^− 3^ substitutions/site/year, which demonstrates that GII polymerases are evolving faster than GI polymerases. It is noteworthy that sampling bias could have impacted these observations. Additionally, GII P16 and P31, which are the polymerase types associated with GII.4 viruses demonstrate a slightly higher substitution rate than the average, which is expected as these viruses have a generally higher evolutionary rates compared to non-GII.4 viruses^33,39^.

A major limitation of this study is that our analysis was based on all of the available complete NS7 (RdRp) sequences that included country of origin and the year of sampling, therefore our dataset was biased towards the dominant sequences from developed countries. To overcome this issue, it is required that researchers from high-income countries collaborate with colleagues in developing countries to study the epidemiology of norovirus in these regions and deposit the sequences in accessible databases.

Recombination analysis was not conducted because only ORF1 (RdRp) sequences were obtained. Established approaches for norovirus recombination detection require paired ORF1–ORF2 sequences spanning the recombination hotspot at the ORF1/ORF2 junction; therefore, recombination could not be reliably assessed for this dataset.

In conclusion, despite extensive genetic diversity among polymerase types, functional motifs remain generally unchanged, reflecting strong purifying selection and essential constraints on replication. GII polymerases exhibit faster evolutionary rates than GI, with P16 and P31 showing slightly elevated substitution rates, consistent with their historical association with pandemic GII.4 strains. The absence of geographic clustering highlights the global dissemination of noroviruses due to high transmissibility. Overall, these findings provide valuable insights into norovirus molecular evolution and support the RdRp as a promising target for antiviral development, given its conserved nature and central role in replication.

## Supplementary Information

Below is the link to the electronic supplementary material.

Supplementary Material 1

Supplementary Material 2

Supplementary Material 3

Supplementary Material 4

## Data Availability

All sequences used in this study were obtained from GenBank. Accession numbers are provided in Supplemental Tables 1 and 2.
